# Development of CD4 T Cell Dependent Immunity Against *N. brasiliensis* Infection

**DOI:** 10.3389/fimmu.2013.00074

**Published:** 2013-03-20

**Authors:** Marina Harvie, Mali Camberis, Graham Le Gros

**Affiliations:** ^1^Queensland University of TechnologyBrisbane, QLD, Australia; ^2^Malaghan Institute of Medical ResearchWellington, New Zealand

**Keywords:** regulation, CD4 Th2, protective immunity, helminth

## Abstract

Of all the microbial infections relevant to mammals the relationship between parasitic worms and what constitutes and regulates a host protective immune response is perhaps the most complex and evolved. *Nippostrongylus brasiliensis* is a tissue migrating parasitic roundworm of rodents that exemplifies many of the salient features of parasitic worm infection, including parasite development through sequential larval stages as it migrates through specific tissue sites. Immune competent hosts respond to infection by *N. brasiliensis* with a rapid and selective development of a profound Th2 immune response that appears able to confer life long protective immunity against reinfection. This review details how the lung can be the site of migrating nematode immune killing and the gut a site of rapid immune mediated clearance of worms. Furthermore it appears that *N. brasiliensis* induced responses in the lung are sufficient for conferring immunity in lung and gut while infection of the gut only confers immunity in the gut. This review also covers the role of IL-4, STAT6, and the innate cytokines IL-25, IL-33, and thymic stromal lymphopoietin in the generation of CD4-mediated immunity against *N. brasiliensis* reinfection and discusses what cytokines might be involved in mediated killing or expulsion of helminth parasites.

## *Nippostrongylus brasiliensis* Infection Induces CD4 T Cell Mediated Th2 Cytokine Immunity

In immune competent rodent hosts primary invasion by *Nippostrongylus brasiliensis* presents as a self limiting infection which is resolved within 9–14 days (Camberis et al., 2003). *N. brasiliensis* initiates infection at the skin site, migrating in a matter of hours through the vasculature to the lungs where maturation occurs. Through exploitation of host mucus/ciliary lung clearance mechanisms, worms are transported out of the lung to be ingested. The L4/L5 become localized in the duodenum where final maturation to adults occurs, leading to reproductive activity and subsequent egg production before the adult worms are expelled. As a consequence of migration through the host *N. brasiliensis* stimulates immune responses at three distinct peripheral sites; skin, lung, and gut which builds to a maximum expression of effector cells and molecules associated with Th2 immunity 9–14 days following infection. This “Th2 cytokine storm” includes the production of cytokines IL-3, IL-4, IL-5, IL-9, IL-10, IL-13 (Conrad et al., 1990; Kopf et al., 1993; Urban et al., 1998; Finkelman et al., 2000, 2004; Holland et al., 2000, 2005; Liu et al., 2002; Min et al., 2004; Mohrs et al., 2005; van Panhuys et al., 2008; Harvie et al., 2010), and the effector mechanisms mediated by IgE antibodies and inflammatory mucus (Lebrun and Spiegelberg, 1987; Katona et al., 1988) and inflammatory cells such as eosinophils (Shin et al., 1997; Daly et al., 1999; Knott et al., 2007), basophils (Conrad et al., 1990; Min et al., 2004), and the newly characterized nuocytes (Neill et al., 2010).

IL-4 has been a Th2 cytokine of interest in *N. brasiliensis* infection since the discovery that disruption of the IL-4 gene prevents Th2 cytokine responses (Kopf et al., 1993). The developments of new technologies such as IL-4 gene reporter mice have enabled careful and thorough investigation of the Th2 immune response elicited by *N. brasiliensis* infection (Hu-Li et al., 2001; Mohrs et al., 2001, 2005). Experiments using the G4/IL-4 reporter mice (Hu-Li et al., 2001), which express green fluorescent protein (GFP) under the control of the IL-4 promoter, have allowed a detailed quantitative analysis of the generation of Th2 immune responses following *N. brasiliensis* infection (van Panhuys et al., 2008; Harvie et al., 2010) to be performed. Interestingly, it was shown that the IL-4 producing T cell response at the skin, lung, and gut sites of infection peaked at a time when parasites had already migrated through the tissue (Harvie et al., 2010) and were at their egg producing adult phase in the gut. It is unknown whether the expulsion of the worms by the Th2 response at this time is a mechanism to aid in egg survival and distribution in the environment or a host protective mechanism to reduce the burden of gut parasites. Our ability to sensitively detect the generation and maintenance of the Th2 immune response *in vivo* has enabled us to undertake a more detailed study of the Th2 immune response in protective immunity against *N. brasiliensis*, and identify the cellular and molecular cues produced by the parasite which coordinate this response.

## Immunity to Reinfection by *N. brasiliensis* Occurs in the Lung

Previous exposure to infection with *N. brasiliensis* induces potent CD4 T cell immunity in the murine host and upon subsequent reinfection with the parasite, results in 90% fewer worms being able to migrate, mature, and develop to adults in the duodenum. Using this reinfection experimental protocol, *N. brasiliensis* has been used as a model for identifying the cells and molecules that regulate CD4 T cell and Th2 dependent protective immunity.

The skin is the initial site of *N. brasiliensis* infection where infective L3 (iL-3) invade and enter the host. Infective L3 are the only stage capable of penetrating through otherwise intact skin. Normally, worms are able to leave the skin infection site and migrate through the vasculature to the lungs. Interestingly, experiments comparing wild type, IL-5 transgenic and IL-5 deficient animals in an ear air pouch model have shown that worms can be trapped and killed in the skin, due to the action of eosinophils (Daly et al., 1999). These results could provide the context for either innate resistance of various mouse strains to primary infection, or represent the effector stage of an overwhelming adaptive immune response. However experiments by Harvie et al. (2010) comparing migration through skin, found no difference in larval migration through naïve or immune skin using a Balb/c model of *N. brasiliensis* infection. Although it should be noted that in natural epicutaneous infection through the skin experimental protocols may reveal a role for the skin site and skin localized immune cells in immune mediated immunity.

Of recent significance is the finding that the lung can be a site of protective immunity against reinfection with *N. brasiliensis*. Normally, iL-3 burst through into the lung parenchyma as early as 16 h post-infection. In a primary infection, the migration to the lung is traumatic for the host as the migrating worms cause damage to the delicate lung tissue (Marsland et al., 2008). This damage can easily be seen by gross pathological analysis as red petechial spots within the lung parenchyma. It is here in the lung that the iL-3 mature into L4 stage parasites before being transported by the lung mucus/ciliary ladder to the esophagus and swallowed.

Although often not considered, there have been a few reported experiments investigating lung immune responses in *N. brasiliensis* infection. Knott et al. (2009) proposed that damage during the lung phase, or pre-lung phase is responsible for reduced worm burdens in FVB/N mice, a strain found to be resistant to *N. brasiliensis* infection. A further study by Harvie et al. (2010) showed that priming of CD4 T cells in the lung tissue was sufficient to confer protection against reinfection with *N. brasiliensis* at both the lung and gut sites. These investigations definitively demonstrate that CD4 and STAT6 dependent responses at the lung site are required for immunity to *N. brasiliensis* as a significant increase in numbers of migrating larvae are recovered from lung tissue upon reinfection with *N. brasiliensis*.

In a large number of previously reported studies, there has been a major focus on investigating immune responses in the gut in the *N. brasiliensis* infection model. These studies have viewed protective immunity as the ability to expel worms from the gut. Gut expulsion is also profoundly dependent on CD4 T cells and STAT6. IL-4 and IL-13 production by immune cells acting on goblet cells to increase mucous secretion and also cause contraction of smooth muscle, leading to the expulsion of the worms (Lawrence et al., 1996; Urban et al., 1998; Voehringer et al., 2006; Neill et al., 2010). IL-5 and IL-9 are also thought to play a role in worm expulsion (Fallon et al., 2002). The newly described innate lymphocyte subtypes have been proposed to have the potential to also affect worm survival through their expression of IL-13 and IL-5. Although the experimental models of *Nippostrongylus*, infection in RAG1 deficient mice would suggest that nuocytes are not able to directly mount a protective response to *N. brasiliensis* by themselves, they could represent a relevant effector arm of the CD4 T cell response playing a key role in gut immunity (Neill et al., 2010).

In conclusion it has become appreciated that although Th2 cells get generated or differentiate in the lymph nodes following parasite infection or allergen challenge their subsequent sequestration to tissues such as the lung, gut and skin can lead to them developing potentially different types of activation dynamics and functional phenotype (Harris et al., 2002). Although this functional plasticity is much better appreciated in studies of CD8 T cells and viral infections, new data and models are emerging for CD4 T cells and innate cells in the context of helminth infections and allergic responses. The studies reviewed above point to the lung being a key tissue involved in generating and being a site of Th2 immune responses. In experimental models the Th2 responses in the lung appear able to significantly arrest the development and function of helminth parasites through the CD4 T cell mediated production of cytokines and effector molecules.

## Immunity to *N. brasiliensis* Reinfection is Generated in the Lung

Infection with helminths such as *N. brasiliensis* presents a unique challenge to the immune system in that over a period of several days the parasite moves to quite different tissue sites including, dermis, blood, lung, and duodenum and at each of these sites presents as an antigenically and biologically different form. The CD4 T cell response that mediates protection against reinfection could be generated at either one of these immune sites or the same one at all sites. Our recent study of *N. brasiliensis* infection in G4/IL-4 reporter mice attempted to determine which site is the most relevant to generating the Th2 cells which mediate immunity against this parasite (Harvie et al., 2010). Surprisingly, these studies found in an intradermal infection model that the skin is not a site of immune protection or immune priming for protection against *N. brasiliensis* infection. These conclusions were drawn from studies where the primary immune response to worms was induced by injecting dead worms in the skin which obviously could not migrate to other tissue sites and prime Th2 responses elsewhere and these did not confer protection against reinfection in the lung (Harvie et al., 2010). However, it could be imagined that under special circumstances of high infection rates or vaccination boosting that skin infection could lead to generation of protective immunity in the lung (Daly et al., 1999; Girod et al., 2003; Fujiwara et al., 2006; Knott et al., 2007). One point to note is that these protocols bypassed the epicutaneous infection stage of worm infection by direct injection into the dermis and it may be that immunity could be both generated and effected at this site if a natural infection model of epicutaneous infection through the skin was employed.

In the same study using an experimental protocol where priming of CD4 T cells against *N. brasiliensis* was confined to gut tissues using gavaged L5/adult worms lung protective immunity against *N. brasiliensis* was not generated. However, mice whose immune priming was confined to the lung site via intranasal priming with L3/L4 stage *N. brasiliensis* were protected at both the lung site and also the gut. Using the G4 reporter mice which revealed the priming of CD4+ T cells it could be shown that this generation of protective immunity was closely associated with the appearance of significant numbers of IL-4 producing CD4 positive cells. Collectively, these results demonstrated that CD4 T cell mediated protection against reinfection could be conferred when animals were primed at the lung site.

An interesting possibility is that the Th2 response in the lung modifies the environment of the lung so that it is no longer supportive of *N. brasiliensis* development. Experiments by Reece et al. (2008) investigate the impact of *N. brasiliensis* infection on the lung environment using mRNA transcript level analysis of cytokines found that *N. brasiliensis* infection induces the development of alternately activated macrophages (AAMs) in a STAT6 dependent manner. This finding builds on the earlier work of Marsland et al. (2008) who also investigated lung pathology and saw the induction of alternately AAMs after *N. brasiliensis* infection. Both of these groups focused on the physical pathological changes in the lung and related the CD4 T cell role in activating AAMs to the ongoing repair of tissue damage, it remains to be determined whether they may also mediate an anti-parasite role upon reinfection.

## The Cells, Cytokines, and Signals Required for Generating CD4 T Cell Mediated Immunity Against *N. brasiliensis* Reinfection

The most compelling argument for the role of CD4 T cells in mediating protective immunity against *N. brasiliensis* infection comes from studies with MHC class II deficient and STAT6 deficient mice. When infected, these immune deficient mouse strains fail to mount Th2 effector responses, fail to clear worms in primary infection and lose the ability to prevent reinfection. In immune competent mice the protective CD4 T cell immunity appears to last for at least 9 months following primary *N. brasiliensis* infection. The observation that CD1 deficient mice show no ablation of protective immunity would suggest that there is no major role for NKT cells in immunity against *N. brasiliensis*. Infection studies with B cell deficient mice reveal perhaps somewhat surprisingly, that antibodies play little role, despite the often-reported association between high IgE levels and immunity to parasites.

The attempts of investigators to identify in more satisfactory detail, which elements of a Th2 mediated STAT6 dependent responses confer immunity have proven elusive to date. It is well established that the cytokine IL-4 is necessary for protection against *N. brasiliensis*, however it does not appear to be linked to its role in driving IgE production but rather a mechanism also able to be mediated by IL-13. Although the exact effector mechanisms employed by Th2 cell mediated protective immunity remain undefined, it has been recently demonstrated that a diverse T cell receptor repertoire is required for protective immunity at the gut site of infection (Seidl et al., 2011). This finding would imply that simply having high numbers of effector cells in the locality of a parasite is insufficient and that intimate MHC restricted, CD4 T activation is also required for conferring protection.

## Role of Innate Cytokines and Innate Helper Cell Types in Th2 Immunity

An intriguing feature of the immune response induced by *N. brasiliensis* infection is the selective and specific induction of Th2 cells producing a “cytokine storm” involving all the Th2 cytokines. As a consequence the *N. brasiliensis* infection model has become the “de rigueur” physiological model for evaluating the upstream mechanisms for the induction and regulation of Th2 cells. The recent identification and cloning of candidate innate cytokines either produced in the skin or identified by their gene sequence homology to other CD4 T cell activating factors has given this research field many candidates to evaluate and test with thymic stromal lymphopoietin (TSLP), IL-25, and IL-33 perhaps being the most pertinent candidates. We would argue that to date the results are still somewhat contradictory and much still remains to be done. As an example although TSLP induced DCs have been shown to initiate Th2 immune responses *in vitro* (Sokol et al., 2008), TSLP deficient mice have unimpaired protective responses against *N. brasiliensis* (Massacand et al., 2009). Similarly, recent data appears to suggest a key role for the Th2 cytokine IL-25 in respect to induction of Th2 responses and Th2 mediated worm expulsion (Fallon et al., 2006; Neill et al., 2010). However, the recent discovery of the effect of IL-25 on innate cell lineages of nuocytes (Neill et al., 2010), innate type 2 helper (ih2) cells (Price et al., 2010), and multipotent progenitor type 2 cells (Saenz et al., 2010) to produce Th2 cytokines such as IL-13 now suggest the view that IL-25 has an accessory role in augmentation of a CD4 Th2 response. In considering the role of IL-25 in CD4 T cell mediated protective immunity against *N. brasiliensis* protective immunity several points emerge. Although IL-25 may feature in *N. brasiliensis* expulsion it is independent of T cell mechanisms as injection of IL-25 into RAG deficient mice leads to the expulsion of adult worms (Fallon et al., 2006; Price et al., 2010). In addition, induction of nuocytes and ih2 cells can be achieved in the presence of IL-33 (Neill et al., 2010; Price et al., 2010), but this cytokine is not required to mount a protective response against *N. brasiliensis* (Senn et al., 2000). Moreover, recent studies indicate that IL-33 can drive CD4-mediated IL-13 production in the *N. brasiliensis* infection model although the majority of the effect is at the level of IL-33 induced ILC2 producing IL-13 in the gut environment leading to worm expulsion (Hung et al., 2012).

An interesting set of studies recently resurrected the issue that innate cells able to produce IL-4 could play a role in Th2 differentiation (Le Gros et al., 1990). Specifically, the recent studies identified that IL-4 producing basophils also expressed Class ll MHC antigens and associated machinery for antigen processing and thus were capable of driving Th2 differentiation (Perrigoue et al., 2009). However, in the setting of *N. brasiliensis* infection Th2 immune responses were found to be unimpaired in the absence of basophils (Kim et al., 2010). These seemingly disparate observations highlight the dearth of knowledge that is critical to our understanding of the innate cellular and cytokine mechanisms driving CD4 T cell mediated protective immunity in helminth infections. In conclusion, to date, no conclusive mechanism linking innate cytokines or innate cells to the *in vivo* differentiation and regulation of Th2 cells and CD4 T cell mediated protective immunity has yet to emerge.

## Identification of Th2 Effector Mechanisms Involved in Immunity Against Helminths

Evidence reviewed here shows there are differences in the parameters of lung and gut immunity against *N. brasiliensis*. Using the G4/IL-4 reporter mice it could be shown that this generation of protective immunity was closely associated with the appearance of significant numbers of IL-4 producing CD4 positive cells. A requirement for IL-4 in lung protection that is not necessary for protection within the gut has been shown (Harvie et al., 2010). This may be a reflection of the redundancy in the roles of IL-4 and IL-13 in gut expulsion of worms, where the as yet unknown mechanisms of protection within the lung must be more dependent on IL-4. The role of eosinophils and IL-5 remains unclear, with some reports suggesting eosinophilia is critical for parasite clearance (Shin et al., 1997; Daly et al., 1999; Knott et al., 2007) and others finding that IL-5 deficiency did not affect infectivity (Harvie et al., 2010). These differences could be due to the use of different mouse model systems, but the role of eosinophils remains unresolved.

Other studies looking at protective mechanisms able to affect *N. brasiliensis* have led to the discovery of novel cell types and molecules; in particular a role for RELMβ has been put forward (Herbert et al., 2009) with RELMβ expression coincident with cytokine production and expulsion of *N. brasiliensis* from the gut (Artis et al., 2004).

The recently discovered class of innate lymphocytes such as the nuocyte has also been shown to play a role in IL-13 mediated expulsion of *N. brasiliensis* from the gut (Neill et al., 2010), although their activity is still dependent on CD4 T cells being maintained at this tissue site. The significance of these innate cells in a *N. brasiliensis* infection is unclear as studies in RAG1 deficient mice that have strong innate immune responses, but defective adaptive response, are unable to clear infection, arguing that innate cells have roles only within the context of CD4 T cell mediated responses.

## Summary

In summary, the regulation of CD4 T cell mediated protective immunity against the parasite *N. brasiliensis* is necessarily complex due to the multiple larval stages and different tissues that are involved during migration through the rodent host. There are increasing numbers of studies that seek to tease apart the CD4 T cell components of this fascinating process. Of particular interest is that the development of long-lived CD4 T cell mediated Th2 effector responses are induced at specific tissue sites of infection by the migrating parasite leading to subsequent Th2 mediated effector responses that can confer local immunity at that tissue site. Also it appears some mucosal sites are linked as indicated by the observation that immune priming to migrating worms in the lung was sufficient to subsequently confer CD4 T cell mediated immunity in the gut. The development and maintenance of protective immunity varies by tissue and/or larval stage of the *N. brasiliensis* parasite, with the requirements of immunity being different within lung and gut tissues (Harvie et al., 2010; Neill et al., 2010). At the gut site it seems that while innate type 2 responses have an important role to play in worm expulsion (Fallon et al., 2006; Voehringer et al., 2006; Neill et al., 2010), it appears that the maintenance of these responses are still dependent on CD4 T cells (Neill et al., 2010). Although the Th2 responses mediating protective immune responses at the lung site are less well defined it is clear that activated CD4 T cells can have a key role in protecting against reinfection or reducing significantly the worm burden carried by the host (Harvie et al., 2010).

Identification of the processes, cells and cytokines that regulate the Th2 responses to tissue parasites such as *N. brasiliensis* is proving exciting but controversial. The overexpression transgenic studies of the cytokines IL-33, TSLP, and IL-25 reveal a potential role for each cytokine while the corresponding reverse genetics gene deletion studies reveal either no role or redundant roles in the models of *N. brasiliensis* infection. The role of innate cells such as basophils able to present antigen, produce IL-4 and drive Th2 development has recently challenged the current paradigm that all antigen presentation to CD4 T cells must go via the dendritic cell. However the relevance of this pathway to *N. brasiliensis* infection induced Th2 responses has yet to be confirmed. The recent identification of the other innate lymphocyte subsets such as nuocytes and ih2 that may be able to regulate CD4 T cells has not yet been fully developed. The discovery or correct assignment of other cytokines and effector molecules to the so-called Th2 subset of CD4 T cell responses is an area of active investigation. It is hoped future studies will be able to unravel this fascinating and highly involved process that is relevant to all vertebrate species.

## Conflict of Interest Statement

The authors declare that the research was conducted in the absence of any commercial or financial relationships that could be construed as a potential conflict of interest.
